# Bioinformatic Analysis of Nematode Migration-Associated Genes Identifies Novel Vertebrate Neural Crest Markers

**DOI:** 10.1371/journal.pone.0103024

**Published:** 2014-07-22

**Authors:** Seung-Hae Kwon, Ok Kyu Park, Shuyi Nie, Jina Kwak, Byung Joon Hwang, Marianne E. Bronner, Yun Kee

**Affiliations:** 1 Korea Basic Science Institute Chuncheon Center, Chuncheon, Korea; 2 Division of Biology 139-74, California Institute of Technology, Pasadena, California, United States of America; 3 Department of Systems Immunology, Kangwon National University, Chuncheon, Korea; 4 Department of Molecular Bioscience, College of Biomedical Science, Kangwon National University, Chuncheon, Korea; 5 Institute of Bioscience and Biotechnology, Kangwon National University, Chuncheon, Korea; Academia Sinica, Taiwan

## Abstract

Neural crest cells are highly motile, yet a limited number of genes governing neural crest migration have been identified by conventional studies. To test the hypothesis that cell migration genes are likely to be conserved over large evolutionary distances and from diverse tissues, we searched for vertebrate homologs of genes important for migration of various cell types in the invertebrate nematode and examined their expression during vertebrate neural crest cell migration. Our systematic analysis utilized a combination of comparative genomic scanning, functional pathway analysis and gene expression profiling to uncover previously unidentified genes expressed by premigratory, emigrating and/or migrating neural crest cells. The results demonstrate that similar gene sets are expressed in migratory cell types across distant animals and different germ layers. Bioinformatics analysis of these factors revealed relationships between these genes within signaling pathways that may be important during neural crest cell migration.

## Introduction

Cell migration is essential for tissue and organ formation during embryogenesis and for the regeneration of some adult tissues. Abnormal regulation of cell migration often results in severe developmental defects and can lead to cancer metastasis. The neural crest is a stem cell-like, multipotent, migratory cell population unique to vertebrate embryos. Neural crest progenitors are born at the border of the neural plate and non-neural ectoderm, become localized within the dorsal neural tube and then migrate away from the neural tube as it closes. This emigration process occurs via an epithelial-to-mesenchymal transition (EMT) such that these ectodermally-derived cells delaminate from the dorsal neural tube and invade the surrounding mesenchyme. They then migrate to distant sites in the periphery of embryos and differentiate into various tissues [1]–[4].

Although neural crest development has been extensively studied for several decades, few genes functionally involved in neural crest cell migration have been identified. This is mainly because the neural crest is a vertebrate-specific cell type and it remains impractical to carry out large scale genetic screening in vertebrates due to their relatively long generation times and high upkeep costs. In contrast, invertebrate genetic screens using the nematode *Caenorhabditis elegans* and fruit fly *Drosophila melanogaster* have identified large number of genes involved in key developmental processes. Mutations disrupting cell migration in different cell types have been identified in *C. elegans*, many of which have yet to be fully functionally characterized [5]–[7].

Genome-wide sequencing of many organisms has provided insight into the infrastructures of their genomic organization. To date, a tremendous amount of sequence information exists in available databases and is ripe for bioinformatic analysis. Comparative genomics is a powerful tool for the identification of novel genes associated with conserved biological processes even between evolutionarily distant animal model systems. We previously used this approach to show that genes important for the migration of nematode hermaphrodite-specific neurons (HSNs) are also involved during neural crest development (Kee et al., 2007). Of fifteen identified vertebrate homologs of genes required for HSN neuronal cell migration, thirteen were shown to be expressed in premigratory or migratory neural crest cells [8], demonstrating that many cell migration genes are evolutionary conserved and ancient. The remarkable conservation of genes involved in long-range cell migration between worm HSN and chick neural crest emphasizes the utility of exploiting existing sequenced genomes and information from traditional genetics to identify candidates involved in stereotypic cell biological processes like cell migration.

Here, we ask whether such high conservation also exists for regulatory genes functioning in cell migration processes between migratory nematode cells arising from different germ layers and vertebrate neural crest. To address this, we selected twenty-five *C. elegans* genes required for the migration of various cell types and performed genomic scanning to search for putative vertebrate orthologs; further candidates were identified by bioinformatic analysis of common functional pathway components. This was followed by expression analysis of the putative candidate genes, which revealed previously unidentified regulatory and signaling genes that may play key, conserved roles in controlling cell migration.

## Materials and Methods

### Computational identification of vertebrate orthologs of worm cell migration genes

Twenty-five nematode (*C. elegans*) genes previously identified in genetic screens as essential for migration of various cell types were selected for this study (Table 1) [6], [7]. Putative chicken (*Gallus gallus*) orthologs of the worm genes were identified by comparative genomic scanning, as shown previously [8]. As the chicken genome has been almost completely sequenced, we were able to identify putative orthologs corresponding to all twenty-five *C. elegans* cell migration genes (Table 1).

**Table 1:** Putative vertebrate orthologs of *C. elegans* cell migration genes in neural crest development.

C. elegans gene	C. elegans cell type	Vertebrate ortholog	Type of encoded protein	Similarity	EST clone	pNCC (NF)	mNCC MB HB
***cam-1***	CAN, ALM neuron, QR, HSN [19]–[21]	MUSK	Receptor tyrosine kinase	4e^−85^	ChEST791o4	−	− −
***ced-2***	DTC [22]	CRK	SH2/SH3 adapter	9e^−25^	ChEST865i8	+	+ −
***ced-5***	DTC [23]	DOCK180	Crk ligand	0	ChEST749l17	+	+ +
***ced-10***	DTC [22]	RAC1	Small rho-like GTPase	1e^−90^	ChEST175j1	+	+ +
***ceh-10***	CAN [24]	CHX10	Homeodomain protein	5e^−49^	ChEST713l20	+	− −
***daf-12***	DTC [25]	NR1I3	Nuclear hormone receptor	3e^−22^	ChEST150a23	+	+ +
***egl-15***	SMs [26]	FGFR1	Growth factor receptor	1e^−113^	ChEST301o3	+	+ +
***egl-17***	SMs [27]	FGF18	Growth factor	0.001	ChEST352a15	+	+ −
***fbl-1***	DTC [28]	FBLN2	Basal membrane protein	7e^−96^	ChEST56a7	−	+/− −
***gon-1***	DTC [29]	ADAMTS9	Metalloprotease	0	ChEST666d18	−	− −
***hch-1***	QL [30]	MEPRIN A, alpha	Metalloprotease	3e^−27^	ChEST115d13	−	− −
***lin-17***	QL [31]	FRIZZLED10	Wnt receptor	5e^−83^	ChEST438c8	+	− −
***lin-39***	QR [32]	HOXA4	Transcription factor	3e^−24^	ChEST427p4	+	− −
***mab-5***	QL, male SMs [33]	HOXB6	Transcription factor	1e^−22^	ChEST147l22	+	+ +
***mig-8***	DTC [25]	Cytochrome P450 2k1-like	Fatty acid hydUlk2 roxylase	4e^−60^	ChEST146o18	−	− −
***mig-13***	QL, QR [34]	Unknown	Transmembrane protein	2e^−07^	ChEST770a16	−	− −
***mig-17***	DTC [35]	ADAMTS18	Metalloprotease	6e^−20^	ChEST399l21	−	− −
***mig-23***	DTC [36]	NTPDase4	Nucleoside diphosphatase	8e^−87^	ChEST276a11	−	− −
***sem-5***	SMs [37]	GRB2	SH-2/SH-3 adaptor protein	2e^−68^	ChEST1027k22	+	+ −
***unc-5***	DTC [38], [39]	UNC5B	Netrin receptor	9e^−84^	ChEST63p10	−	− −
***unc-6***	DTC, neurons, linker cell [38], [40]	NTN1	Netrin	0	ChEST1011d23	−	− −
***unc-40***	DTC, SDQR, linker cell, QL daughters [38], [41]	DCC	Netrin receptor	1e^−144^	ChEST741m4	+	− −
***unc-51***	CAN [42]	ULK2	Protein kinase	8e^−73^	ChEST545c22	+	+ −
***unc-53***	ALN & PLN axons, muscles, excretory canals [43]-[45]	NAV2	Unknown	2e^−64^	ChEST724g24	−	− −
***vab-8***	ALM neuron, CAN [46], [47]	KINESIN	Motor protein	1e^−25^	ChEST46k4	+	− +

ALM, anterior lateral microtubule: CAN, canal-associated neurons: DTC, distal tip cells: *mNCC, migratory neural crest cells: NF, neural fold: pNCC, pre-migratory neural crest cells: QL, left Q neuroblasts: QR, right Q neuroblasts: SDQR, a neuron positioned along right lateral side of midbody in C. elegans: SMs, sex myoblasts: +, detected: −, not detected.

### DNA constructs and RNA probe synthesis

Sequences of putative vertebrate orthologs corresponding to all twenty-five *C. elegans* cell migration genes were used as queries to search the chicken EST database at www.chick.umist.ac.uk/ (Table 1). Chicken EST clones of the genes in the predicted FGFR signaling pathway were identified in a similar manner: SOS1 (ChEST793F19), GAB1 (ChEST878L14), FRS2 (ChEST166G11), Shc1 (ChEST352e24), SOS2 (ChEST173l15). PIK3R2 was isolated in our previous screen [9]. Antisense RNA probes for *in situ* hybridization were prepared as previously described [10].

### Embryo collection and whole mount *in situ* hybridization

White Leghorn chicken eggs were obtained from local farms and were incubated at 38°C for two to three days. Embryos were fixed in 4% paraformaldehyde in PBS (Phosphate Buffered Saline) at 4°C overnight, and subjected to whole mount *in situ* hybridization, as previously described [11], [12]. Stages were determined according to criteria of Hamburger and Hamilton [13]. National Institute of Health guidelines state that for chicken embryos younger than embryonic day 10, no special approval or IACUC documentation is required.

### Pathway analysis

Predicted signaling pathways were obtained from our identified putative vertebrate orthologs using Gene Ontology and MetaCore (GeneGo) analysis with the canonical pathways setting. A specific sub-pathway was generated by limiting the connections of the components in each pathway including only genes identified from this study.

## Results and Discussion

### Genomic scanning of cell migration genes

Twenty-five genes, identified from genetic screens in *C. elegans* for their functional importance in cell migration in various tissues [6], [7], were selected for computational analysis. Using BLAST search engines against available vertebrate genome sequences and genomic databases [8], we identified vertebrate counterparts, here defined as putative orthologs. The vertebrate gene with highest identity score by computational analysis was used to identify the putative chick ortholog and its available EST (Expressed Sequence Tags) sequence. In this way, we identified orthologs in the chicken genome corresponding to all twenty-five *C. elegans* cell migration genes (Table 1).

### Expression screening of cell migration genes

**Figure 1:**
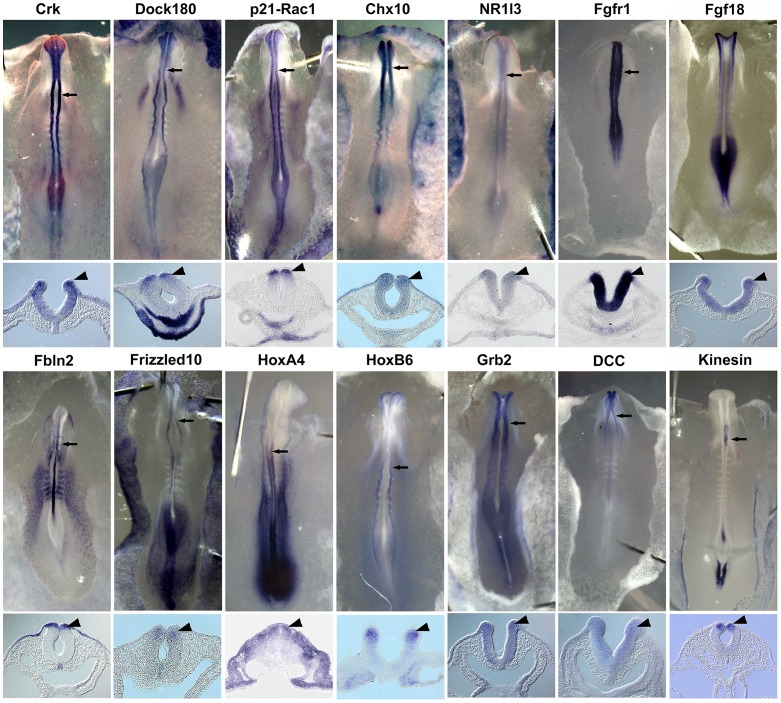
Vertebrate orthologs of genes essential for cell migration in *C. elegans* are expressed in premigratory neural crest cells in chicken embryos. Whole mount *in situ* hybridization was performed using RNA probes corresponding to orthologs of each nematode gene (upper panel). Of the twenty-five genes examined in this study, fourteen chicken orthologs were expressed in the premigratory neural crest domain in the neural folds, as clearly shown in sections (lower panel); Crk (ced-2), Dock180 (ced-5), p21-Rac1 (*ced-10*), Chx10 (*ceh-10*), NR1I3 (*daf-12*), Fgfr1 (*egl-15*), Fgf18 (*egl-17*), Fbln2 (*fbl-1*), Frizzled10 (*lin-17*), HoxA4 (*lin-39*), HoxB6 (*mab-5*), Grb2 (*sem-5*), DCC (*unc-40*) and Kinesin (*vab-8*). arrow, plane of section; arrowhead, gene expression in neural fold.

To test whether the twenty-five vertebrate orthologs of the worm genes were expressed during neural crest development, we performed *in situ* hybridization at various stages beginning at the premigratory stage (HH8) when cranial neural crest cells are located within the dorsal neural tube and have yet to delaminate from the neuroepithelium by undergoing EMT to initiate migration at HH9. Thirteen chicken transcripts, homologous to the *C. elegans* cell migration genes *ced-2* (Crk), *ced-5* (Dock180), *ced-10* (p21-Rac1), *ceh-10* (Chx10), *daf-12* (NR1I3), *egl-15* (Fgfr1), *egl-17* (Fgf18), *lin-39* (HoxA4), *mab-5* (HoxB6), *sem-5* (Grb2), *unc-40* (DCC), *unc-51* (ULK2) and *vab-8* (kinesin), were detected in premigratory neural crest cells in the dorsal neural tube (Figure 1). Neural crest expression was not uniform in every case: the putative vertebrate ortholog of *lin-39* (HoxA4) for example, was only expressed in the neural folds of the caudal trunk at later stages (Figure 1). Furthermore, expression of the *fbl-1* ortholog FBLN2 was limited to the tip of the dorsal neural tube and was also expressed in the cranial ectoderm, while the *vab8* ortholog (kinesin) was expressed only in the dorsal hindbrain. Thus, we identified homologs of nematode cell migration genes that are expressed in subpopulations of premigratory neural crest cells from different axial levels of the neural tube. All of these genes have known functions in cell migration in the nematode (Table 1). The fact that they are also expressed by premigratory neural crest cells as they are preparing to initiate EMT raises the intriguing possibility that they may be functionally important in vertebrates as well.

**Figure 2:**
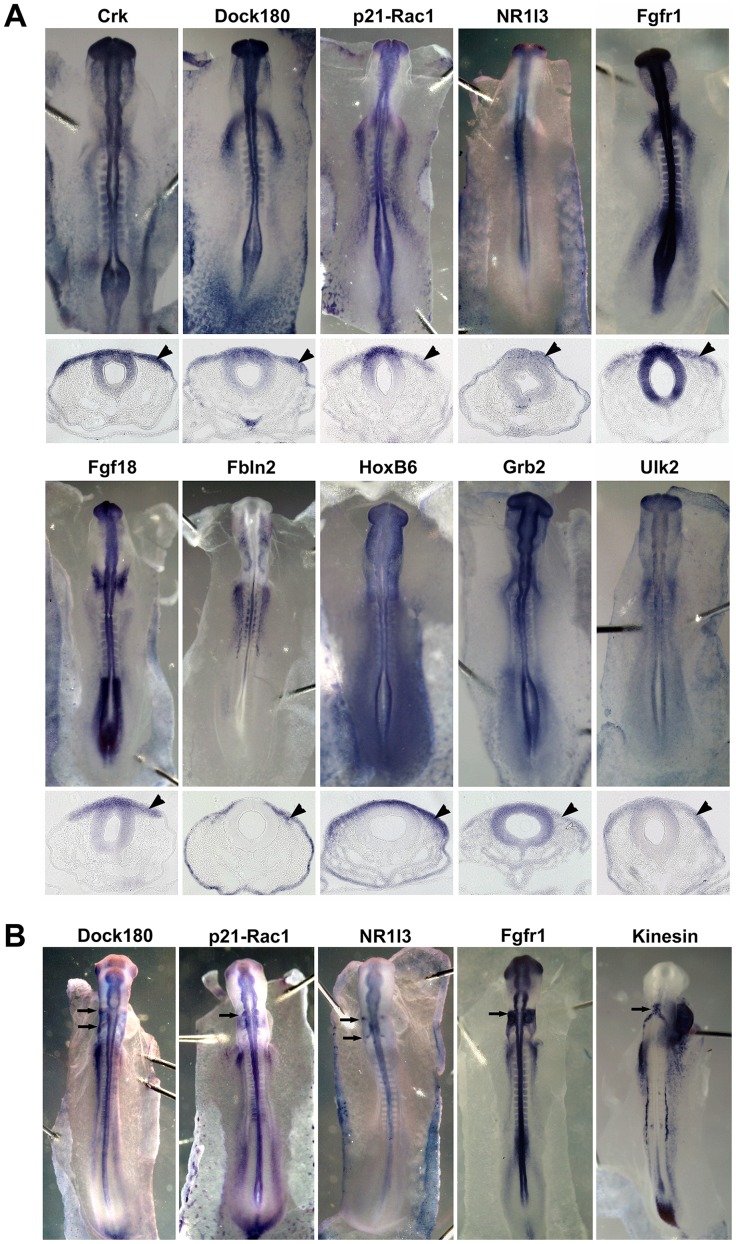
Putative chicken orthologs of nematode cell migration genes are conserved in migrating cranial neural crest cells. Chicken embryos were subjected to whole mount *in situ* hybridization using RNA probes corresponding to the vertebrate orthologs of each nematode gene. (A) Whole mount chicken embryos (upper panel) and tissue sections at the midbrain level of each embryo (lower panel) show that ten vertebrate orthologs are expressed in neural crest cells migrating from the neural tube in chicken embryos at HH stage 9–10; Crk (*ced-2*), Dock180 (*ced-5*), p21-Rac1 (*ced-10*), NR1I3 (*daf-12*), Fgfr1 (*egl-15*), Fgf18 (*egl-17*), Fbln2 (*fbl-1*), HoxB6 (*mab-5*), Grb2 (*sem-5*) and Ulk2 (*unc-51*). Arrowhead indicates migrating neural crest cells. (B) Five vertebrate orthologs are expressed in migrating neural crest cells at the hindbrain level in HH stage 11–13 embryos; Dock180 (*ced-5*), p21-Rac1 (*ced-10*), NR1I3 (*daf-12*), Fgfr1 (*egl-15*) and Kinesin (*vab-8*). Arrows indicate gene expression in migrating cranial neural crest cells in rhombomere 4 and/or rhombomere 6.

At later stages, chick orthologs of *ced-2* (Crk), *ced-5* (Dock180), *ced-10* (p21-Rac1), *daf-12* (NR1I3), *egl-15* (Fgfr1), *egl-17* (Fgf18), *mab-5* (HoxB6), *sem-5* (Grb2) and *unc-51* (Ulk2) were expressed in migrating neural crest cells at the midbrain level (Figure 2A). The ortholog of *fbl-1* (FBLN2) was transiently expressed in a small population of migrating cells and in the ectoderm (Figure 2A). Finally, the vertebrate orthologs of *ced-5* (Dock180), *ced-10* (p21-Rac1), *daf-12* (NR1I3), *egl-15* (Fgfr1), *mab-5* (HoxB6) and *vab-8* (kinesin) were detected in migrating neural crest cells at the hindbrain level of stage 11–13 embryos (Figure 2B, mab-5 in Figure 1). Interestingly, the vertebrate ortholog of *vab-8*, kinesin, was specifically expressed in a subset of premigratory neural crest cells in rhombomeres 4 through 6 (r4 to r6) and in the r6 migration stream in the hindbrain (Figure 2B).

Cumulatively, the chick orthologs of nematode migratory genes expressed in the neural crest include two transcription factors, HoxA4 (*lin-39*) and HoxB6 (*mab-5*), eight signaling molecules including Crk (*ced-2*), Dock180 (*ced-5*), Chx10 (*ced-10*), p21-Rac1 (*ceh-10*), HoxA4 (*lin-39*), HoxB6 (*mab-5*), Grb2 (*sem-5*) and Ulk2 (*unc-51*), three growth factors or receptors, Fgfr1 (*egl-15*), Fgf18 (*egl-17*) and Frizzled10 (*lin-17*), a single nuclear hormone receptor NR1l3 (*daf-12*), a guidance molecule DCC (*unc-40*), a basal membrane protein Fbln2 (fbl-1), and a motor protein Kinesin (vab-8) (Table 1). These findings suggest that the expression of a variety of molecules important for long-range cell migration in nematode, including transcription factors, are common to migratory cells in vertebrates as well. Thus, they may have general roles in EMT and/or motility, regardless of their tissue origins. This comparative analysis revealed that 56% of the vertebrate orthologs of C. *elegans* genes were expressed in neural crest cells, contrasting with 87% for HSN genes in our previous study. This suggests that cells originating from different germ layers may share fewer orthologous migratory genes than those from the same germ layer.

### Functional pathway analysis

**Figure 3:**
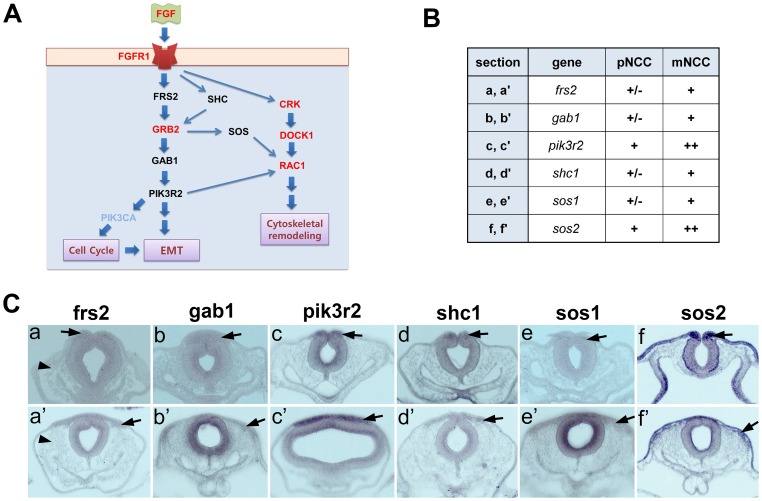
Functional pathway analysis. (A) Predicted FGFR signaling pathway map showing genes obtained from our comparative analyses in red. Whole mount *in situ* hybridization using RNA probes corresponding to the genes in black was performed. (B) Table summarizing gene expression in neural crest cells. pNCC, premigratory neural crest cells: mNCC, migratory neural crest cells: -, not detected: +, detected: ++, expressed at high level. (C) Transverse sections of chick embryos showing gene expression in premigratory and/or emigrating neural crest cells at stage HH 8–9 (a–f) and in migrating neural crest cells at the midbrain level at HH stage 9–10 (a′–f′); (a, a′) frs2, (b, b′) gab1, (c, c′) pik3r2, (d, d′) shc1, (e, e′) sos1, (f, f′) sos2. Arrows indicate emigrating neural crest cells (a–d, f) or premigratory neural crest cells (e). Arrowheads indicate head mesenchymal cells (a,a′).

To identify the biochemical pathways in which the identified vertebrate orthologs might act, we performed bioinformatic analysis using MetaCore (GeneGo) software and The Gene Ontology Consortium. This provided candidate pathways in which the orthologous genes had been previously implicated. One of the best hits was in the developmentally-related FGFR signaling pathway, a canonical pathway in the MetaCore database. By analyzing genes common to this pathway, we extracted a predicted pathway including direct connections with the genes identified from our expression studies; FGF, FGFR1, GRB2, CRK, DOCK1 and RAC1 (Figure 3A, red). The molecular components of this map were previously implicated in EMT and cytoskeletal remodeling during cell migration [14]–[16].

To determine whether the members of this predicted pathway are conserved in migrating neural crest cells, the remaining pathway components (Figure 3A, black letters) were analyzed by *in situ* hybridization. Our results show that transcripts of FRS2, GABA1, SHC1, and SOS1 are barely detectable in premigratory neural crest cells (Figure 3C a, b,d,e), but are present at low levels in migrating neural crest cells (Figure 3C a′, b′,d′,e′). The guanine nucleotide exchange factors son of sevenless 1 (SOS1) and son of sevenless 2 (SOS2) function in multiple signaling pathways and have been implicated in a wide variety of biological and oncogenic processes [17]. Interestingly, SOS2 is highly up-regulated in the emigrating neural crest cells and maintained in migrating neural crest cells (Figure 3C, f,f′), while SOS1 is expressed in migrating neural crest cells at low levels (Figure 3C e′). PIK3R2 (p85β phosphoinositide 3-kinase subunit) is known to regulate tumor progression: p85β expression is elevated in breast and colon carcinomas and genetic alteration of PIK3R2 expression levels modulate tumor progression in vivo [18]. Our result shows PIK3R2 is up-regulated in the neural crest cells emigrating out of neural tube (Figure 3C, c,c′) and in migrating neural crest cells [9]. Our functional pathway analysis suggests that up-regulation of PIK3R2 and SOS2 correlate with neural crest EMT and that SOS2 appears to be more prevalent than SOS1 in early neural crest development. FGF signaling is known to be required for neural crest induction or specification from neural stem cells, but has not been previously implicated in neural crest EMT or initial cell migration. Thus, our genomic and pathway analysis have revealed previously unidentified genes and pathways that may be critical for normal emigration and/or migration of neural crest cells and are promising candidates for future functional experiments.

## Conclusions

Large quantities of genomic sequencing data are available in diverse databases that are ripe for mining. Genetic screens in invertebrates such as worms and fruit flies have identified many molecules important in behavioral, developmental, and physiological processes. Here, we have broadened the utility of cross-species comparative genomic screening by combining it with functional pathway analysis to identify additional candidate genes for analysis. Our results have uncovered previously unidentified molecular players up-regulated during the course of neural crest migration. It is interesting to note that many of the genes identified have previously been assumed to be ubiquitously expressed, and would be unlikely to be identified by conventional screens. This proof-of-principle study validates our combinatorial strategy as an effective pipeline for the identification of novel regulatory genes during neural crest development and other complex migratory processes.
